# Effect of Municipal Solid Waste Incineration Fly Ash on the Mechanical Properties and Microstructure of Geopolymer Concrete

**DOI:** 10.3390/gels8060341

**Published:** 2022-05-30

**Authors:** Mengya Niu, Peng Zhang, Jinjun Guo, Jia Wang

**Affiliations:** School of Water Conservancy Engineering, Zhengzhou University, Zhengzhou 450001, China; niumengya00@126.com (M.N.); jinjunguozzu@163.com (J.G.); wangjiasf@163.com (J.W.)

**Keywords:** municipal solid waste incineration fly ash, geopolymer concrete, mechanical properties, fracture properties

## Abstract

Geopolymers are environmentally friendly materials made from industrial solid waste with high silicon and aluminum contents, and municipal solid waste incineration fly ash (MFA) contains active ingredients such as Si, Al and Ca. According to this fact, a green and low-carbon geopolymer concrete was prepared using MFA as a partial replacement for metakaolin in this study. The mechanical properties of the MFA geopolymer concrete (MFA-GPC) were investigated through a series of experiments, including a compressive strength test, splitting tensile strength test, elastic modulus test and three-point bending fracture test. The effect of the MFA replacement ratio on the microstructure of MFA-GPC was investigated by SEM test, XRD analysis and FTIR analysis. MFA replacement ratios incorporated in GPC were 5%, 10%, 15%, 20%, 25%, 30%, 35% and 40% by replacing metakaolin with equal quality in this study. In addition, toxic leaching tests of MFA and MFA-GPC were performed by ICP-AES to evaluate the safety of MFA-GPC. The results indicated that the mechanical properties of MFA-GPC decreased with the increase of the MFA replacement ratio. Compared with the reference group of GPC without MFA, the maximum reduction rates of the cubic compressive strength, splitting tensile strength, axial compressive strength, elastic modulus, initiation fracture toughness, unstable fracture toughness and fracture energy of MFA-GPC were 83%, 81%, 78%, 93%, 77%, 73% and 61%, respectively. The microstructure of MFA-GPC was porous and carbonized; however, the type of hydrated gel products was still a calcium silicoaluminate-based silicoaluminate gel. Moreover, the leaching content of heavy metals from MFA-GPC was lower than that of the standard limit. In general, the appropriate amount of MFA can be used to prepare GPC, and its mechanical properties can meet the engineering requirements, but the amount of MFA should not be too high.

## 1. Introduction

In the wake of the rapid development of urbanization and industrialization, the demand for building materials is increasing every year [1]. Concrete is the most basic artificial construction material in the world [2,3]. Because of its excellent mechanical properties, durability, availability of constituent materials and good plasticity, concrete is considered to be the most commonly used building material [4,5]. However, the production of ordinary Portland cement (OPC) concrete requires a lot of energy and produces a lot of carbon dioxide [6,7,8,9]. Chinese production of cement reached up to 2362 million tons in 2021 [10]. As a major greenhouse gas, carbon dioxide is responsible for the severe phenomenon of global warming [11], while producing 1 ton of Portland cement will produce approximately 1 ton of carbon dioxide gas [12]. Geopolymer is a new cementitious material with high silicon and aluminum contents and a three-dimensional Si-O-Al polymeric network structure composed of tetrahedral units [13,14]. Various combinations of mixing and processing parameters are considered to have a critical impact on geopolymers [15]. Abadel et al. [16] found that the molar ratios had an effect on the strength, microstructure and embodied energy of geopolymer. They point out that the molar ratio of silica to alumina should be greater than 2.3 to produce a high-strength structure. Geopolymer is formed by alkali excitation with natural silicon aluminum compounds or industrial solid wastes [17,18,19]. Because the raw materials of geopolymer are majorly industrial solid wastes, e.g., fly ash [20], slag [21], waste glass [15] and steel slag [22], it can effectively realize the sustainable utilization of natural resources as a promising alternative to cement [23]. In addition, previous study results have shown that geopolymer can effectively seal Pb, Zn, Cd and other heavy metal ions by chemical bonding and physical fixation [24]. By using industrial by-product waste as a raw material, geopolymer concrete (GPC) can reduce CO_2_ emissions by up to 80% compared with OPC concrete [25]. Many scholars have studied the formation mechanism and internal structure characteristics of geopolymer, and carried out a series of research on the mechanical properties of GPC prepared with geopolymer as cementitious material. GPC is an environmentally friendly material, first introduced in 1975 by Davidovits [26]. Under a similar compressive strength, GPC has the characteristics of quick hardening and a better bonding strength compared with OPC concrete [22].

Owing to the rapid growth of municipal solid waste (MSW) production and the lack of land for landfill treatment, incineration is considered worldwide to be the most advanced technology for the disposal of MSW, because it can significantly reduce the volume of the waste (by 90%) [27], reduce risk and recover energy [28,29,30]. Municipal solid waste incineration fly ash (MFA) is produced inevitably during the MSW incineration [31], which accounts for about 3~5% of the total incinerated MSW [32]. It is estimated that more than 3.7–18.3 million tons of MFA are engendered every year in China [33]. The toxic substances contained in MFA such as heavy metals (Pb, Zn, Cd and Cr) are harmful to human health and the surrounding environment [34]. Therefore, treating MFA innocuously and efficiently has attracted global academic attention. Four different common procedures are often used for treating MFA: solidification, stabilization, thermal treatment and separation [35,36]. The cement solidification method is applied to treat the waste material for immobilizing contaminates and usually reduces leachability [35]. However, there are strong limitations regarding the volume of MFA incorporated into cementitious mixes [37]. In addition, heavy metals and high concentrations of salt in the solidified body often lead to the rupture of the solidified body, resulting in heavy metal leaching and secondary pollution [38]. In chemical stabilization, inorganic and organic heavy metal immobilizers are mixed evenly with MFA to convert the heavy metals into insoluble heavy metal chelates. [34]. However, chemical stabilization still requires landfills and the heavy metal immobilizers are expensive. The thermal treatment method is mainly done by melting [39], sintering [40] or vitrification [41]. However, it has not been widely used as some heavy metals with a low boiling point will volatilize into flue gas during the treatment process, resulting in secondary pollution [42]. The metal separation method can extract heavy metals, such as acid extraction and bio-extraction [43]. However, the treatment process is complicated and some heavy metals still remain in the MFA [34]. Therefore, in terms of reliability, economy and environmental safety, the traditional MFA treatment methods are still insufficient. The effective utilization of MFA is conducive to the sustainable development of resources; hence, how to safely and effectively utilize MFA with huge output is a topic actively advocated all over the world.

The chemical constituent of MFA is rich in SiO_2_, Al_2_O_3_, CaO and Fe_2_O_3_ [42,44]. Therefore, many scholars have carried out research on the preparation of MFA-based geopolymer and utilized geopolymer technology to immobilize MFA in recent years [28]. Zhang et al. [45] explored the mechanical properties of MFA-based geopolymer and revealed the mechanism of geopolymer curing heavy metals. The results showed that the compressive strength of MFA-based geopolymer can be effectively promoted by increasing the concentration of alkali activator. Liu and other investigators [46] prepared MFA-based geopolymer with metakaolin and MFA as raw materials, and the properties of the geopolymer were researched. It was revealed that the geopolymer with 10% contents of metakaolin was consistent with the hydration products of pure MFA-based geopolymer. The addition of metakaolin significantly improved the mechanical properties of the geopolymer. The compressive strength of 10% metakaolin geopolymer was twice that of pure MFA-based geopolymer. In order to solve the problem of less silicon and aluminum content in MFA, Ren et al. [47] utilized silica powder to replace MFA to prepare geopolymer. They pointed out that adding silica fume promoted the formation of hydration products such as C-S-H gel, and the morphology of geopolymer doped with silica fume was denser than pure MFA-based geopolymer. Similarly, Tian et al. [48] partially replaced MFA with fly ash and observed that adding fly ash makes the microstructure steady and dense. Li et al. [49] prepared a composite geopolymer with MFA and red mud, and they found that adding excessive MFA will lead to chloride and calcium. Ye et al. [28] also studied a geopolymer prepared from MFA and Bayer red mud, and the results showed that the compressive strength of the geopolymer can be effectively promoted by adding 50–60% Bayer red mud, and most heavy metals such as Zn, Pb and Cu in MFA changed from a leachable state to a leachable residual state. They concluded that MFA had higher silicon and aluminum contents than Bayer red mud, so the participation of MFA increased the SiO_2_/Al_2_O_3_ ratio in the matrix and improved the structural stability of the geopolymer. Jin et al. [50] studied the acid and alkaline resistance of MFA-based geopolymer. The results showed that the compressive strength was 36.1 MPa after acid rain leaching and still reached 40.0 MPa after alkali solution soaking, and the leaching content of heavy metals was lower than the standard limit. Abundant studies showed that most of the geopolymer prepared from MFA, fly ash and metakaolin had excellent working properties, mechanical properties, durability and fixing efficiency of heavy metals [45]. The preparation of MFA-based geopolymer using MFA as the geopolymer precursor has been proved to be a relatively feasible technical method which can effectively solidify heavy metals and realize the resource utilization of MFA.

Recently, the performance of MFA-based geopolymer paste and mortar have been researched [46], while there is a lack of research on MFA geopolymer concrete (MFA-GPC), especially the mechanical performance of MFA-GPC. If MFA-GPC with an excellent working performance, mechanical properties and environmental safety can be prepared by using MFA-based geopolymer as the cementitious material, it can not only provide a new technical route for the resource utilization of MFA, but can also replace partial traditional cement concretes. Therefore, there is a necessity for further research to be performed in this regard. In this study, the effect of MFA content on the mechanical properties of MFA-GPC was investigated, and the influence mechanism of MFA on the microstructure of MFA-GPC was revealed. Using basic mechanical properties tests of MFA-GPC specimens with different MFA contents, the mechanical properties of MFA-GPC were measured. Moreover, the fracture properties of MFA-GPC were studied through three-point bending fracture tests, and the microstructure of MFA-GPC was analyzed by scanning electron microscope (SEM) test, X-ray diffraction (XRD) analysis and Fourier transform infrared spectroscopy instrument (FTIR) analysis. The concentrations of heavy metals in the leaching solution of MFA samples and MFA-GPC specimens were analyzed by inductively coupled plasma-atomic emission spectrometry (ICP-AES).

## 2. Experimental Investigation

### 2.1. Materials

**Figure 1:**
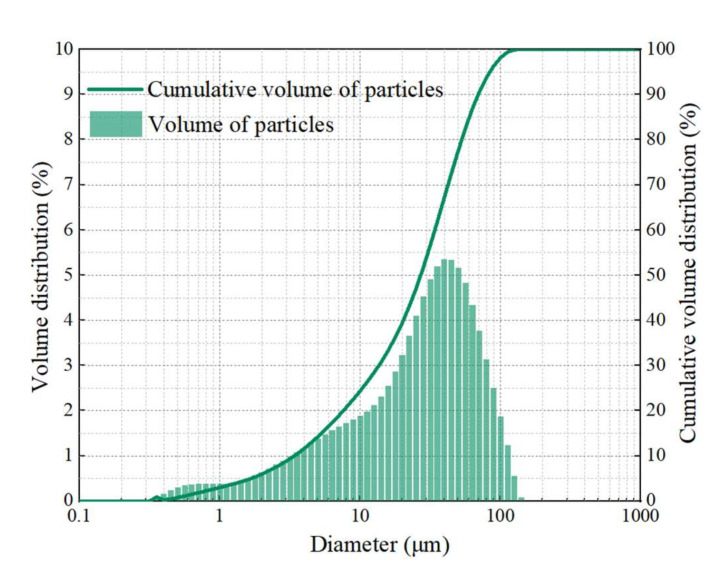
Particle size distribution of MFA.

The MFA was produced from a Luoyang domestic waste incineration plant, and it was dried and screened before use. The particle size distribution of MFA is shown in Figure 1. The main particle size of MFA ranges from 1 to 100 μm, which indicates that MFA has potential gelling activity. High-quality FA was utilized and the metakaolin (MK) was purchased in this study. The chemical compositions of the MFA, FA and MK are presented in Table 1 and Table 2. The sodium hydroxide and sodium silicate were used as alkaline activators, which had a 1.3 molar ratio of silica oxide to sodium oxide, 16.8% weight ratio of sodium oxide, 1.41 specific gravity and 32.4 solid contents. According to the Chinese Standard (GB8076-2008) [51] used for concrete admixture, the superplasticizer (25% of water-reducing rate) was utilized. Coarse aggregates (5–20 mm particle sizes) and river sands (2.7 fineness modulus) were utilized in this study.

### 2.2. Mix Proportions

In this study, mix proportions of MFA-GPC with different MFA replacement ratios were designed according to the Chinese Standard JGJ 55-2011 and related research [52]. The control variable method was adopted in the mix design, in which the water–binder ratio, bone glue ratio, sand ratio, sodium silicate modulus and alkali contents remained unchanged, while the dosage of MFA was changed. The proportion of samples is determined by weight: 3.0 aggregate/binder ratio, 0.35 water/binder ratio and 0.35 sand rate [53,54]. Under the condition that the water–binder ratio remained unchanged, the MFA in this study was mixed into GPC by replacing metakaolin with equal quality, and the dosages were 5%, 10%, 15%, 20%, 25%, 30%, 35% and 40%, respectively. In this study, the dosage of the high-efficiency water-reducing agent was based on the principle of consistency of working performance of each mix proportion, and that its dosage should be decreased with the increase of MFA dosage. The specific mix proportions of MFA-GPC are listed in Table 3.

**Figure 2:**
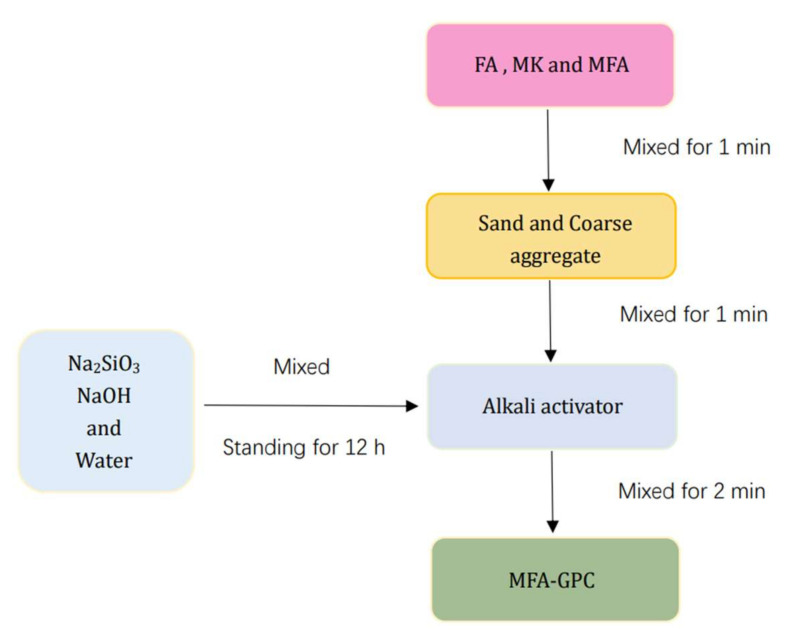
Mixing process of MFA-GPC.

A mechanical agitator with a capacity of 50 L was used to prepare the specimens in this study. The pre-prepared alkali activator and water-reducing agent mixture were poured into the agitator for stirring, and then fresh concretes were loaded into the molds and vibrated to form MFA-GPC specimens. The samples were demolded after 24 h in the forming chamber. After demolding, the specimens were placed in the standard curing room with a temperature of 20 ± 2 °C and a relative humidity of no less than 95% for 28 days. The mixing process of MFA-GPC is shown in Figure 2.

### 2.3. Basic Mechanical Properties Tests

The standard cubic compressive strength and the splitting tensile strength of the MFA-GPC specimens were studied with reference to GB/T 50081-2019 [55]. Three cube specimens with a size of 100 × 100 × 100 mm were prepared for each group. The loading rates were 0.6 MPa/s and 0.06 MPa/s, respectively. The arithmetic means of the compressive strength for the three specimens were taken as the final results for each MFA replacement rate. Prism specimens with dimensions of 100 ×100 × 300 mm were tested according to Chinese national standard GB/T 50081-2019 [55] to research the elastic modulus of the MFA-GPC specimen, and the prism compressive strength was obtained during the course of measuring for the elastic modulus with a loading rate of 0.5 MPa/s. Six prisms were cast for each group to ensure correct results.

### 2.4. Fracture Properties Test

**Figure 3:**
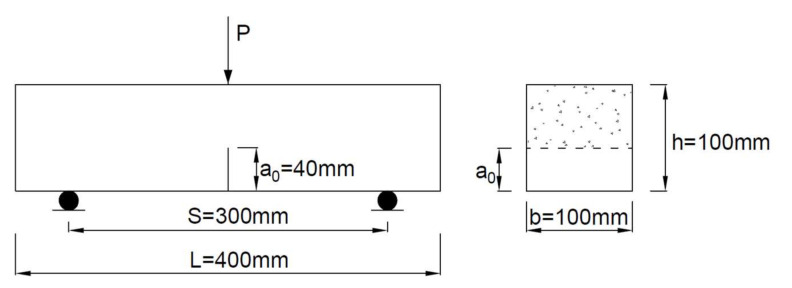
Schematic diagram of specimen for fracture testing.

Three-point bending tests were carried out to evaluate the fracture performance of MFA-GPC. At present, there is no uniform testing standard for MFA-GPC. Therefore, five specimens with sizes of 100 × 100 × 400 mm were used on the basis of the current research work [56,57,58] and the Chinese standard DL/T 5332-2005 [59], which had a 3 × 40 mm initial notch. The average of triplicate specimens was taken as the result for each mixture. The schematic diagram of a specimen for fracture testing is presented in Figure 3.

**Figure 4:**
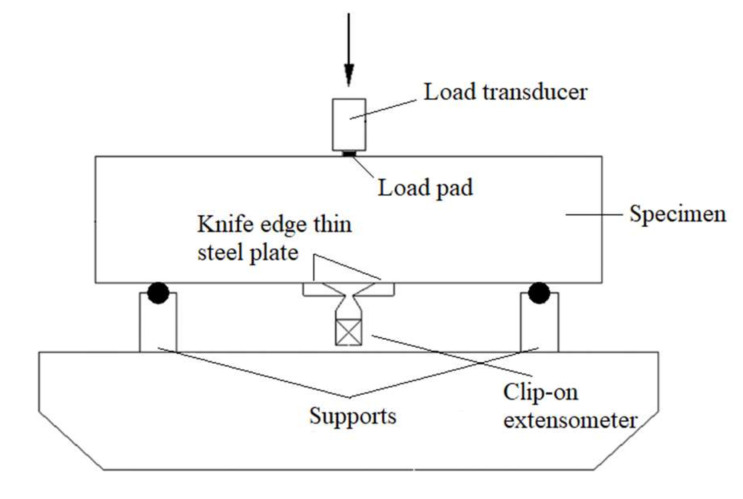
Schematic diagram of the fracture properties test device.

**Figure 5:**
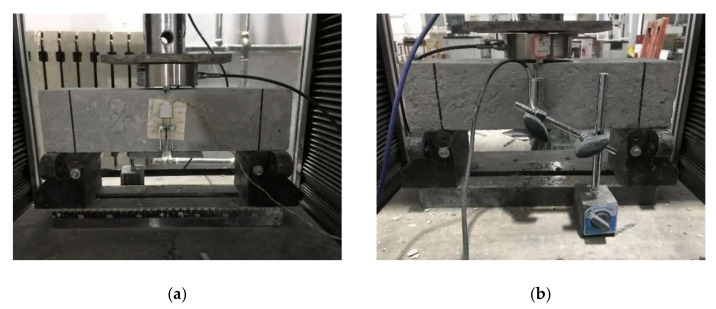
Field diagram of fracture test: (**a**) clip extensometer layout and (**b**) electric displacement meter.

Three-point bending tests were carried out to evaluate the fracture properties of MFA-GPC specimens with different MFA replacement ratios. A WA-1000 microcomputer controlled electrohydraulic service universal testing machine was used as the loading device. A load sensor with a measuring range of 50 kN and a precision of 1 N was utilized as a load acquisition device. An electrical displacement meter with a sensitivity of 0.35 mv/mm was used to measure the mid-span deflection of the specimen. The crack mouth opening displacement (CMOD) of the specimen was measured by a clip gauge (10 mm range, 0.5 mm accuracy) to obtain the CMOD curve. In this study, a DH3818Y static strain tester with an acquisition frequency of 1 Hz was adopted to collect data. The schematic diagram of the fracture properties test device is shown in Figure 4. Thin sheets were affixed to both sides of the prefabricated joint at the bottom of the notched beam specimen for placing the clip-on extensometer. A steel plate was affixed to the center of the bottom of the specimen for placement of the electric displacement meter. A displacement control mode was used with a constant loading rate of 0.1 mm/min. In addition, the load on the specimens was measured by a load sensor. Actual-time loads, crack opening displacement and mid-span deflection changes during the test were collected and stored synchronously by the data acquisition system. The testing apparatus of the fracture properties test is shown in Figure 5.

Based on the double-K fracture theory, the crack initiation load ($F_{Q}$) is the loading point from linear to nonlinear. The initiation fracture toughness ($K_{IC}^{Q}$) can be obtained from Equations (1) and (2):

(1) $$K_{IC}^{Q}=\frac{1.5\left(F_{Q}+\frac{mg}{2}\times 10^{-2}\right)\times 10^{-3}S\sqrt{a_{0}}}{th^{2}}f\left(\alpha_{0}\right)$$

(2) $$f\left(\alpha_{0}\right)=\frac{1.99-\alpha_{0}\left(1-\alpha_{0}\right)\left(2.15-3.93\alpha_{0}+2.7\alpha_{0}^{2}\right)}{\left(1+2\alpha_{0}\right){\left(1-\alpha_{0}\right)}^{3/2}},\;\alpha_{0}=\frac{a_{0}}{h}$$

where $K_{IC}^{Q}$ is the initiation fracture toughness (MPa·m^1/2^); $F_{Q}$ is the crack initiation load (kN); $\alpha_{0}$ is the initial fracture length (0.04 m); h is the specimen height (0.1 m); m is the specimen mass between supports (kg); g represents the gravitational acceleration (9.81 m/s^2^); and S is the supports span (0.3 m).

In the load-CMOD curve, the CMOD corresponding to peak load ($F_{max}$) is the critical CMOD ($V_{c}$). Analogously, the corresponding fracture toughness under the peak load ($F_{max}$) is called unstable fracture toughness ($K_{IC}^{S}$), which can be calculated by Equation (3).

(3) $$K_{IC}^{S}=\frac{1.5\left(F_{\max}+\frac{mg}{2}\times 10^{-2}\right)\times 10^{-3}S\sqrt{a_{c}}}{th^{2}}f\left(\alpha \right)$$

(4) $$f\left(\alpha \right)=\frac{1.99-\alpha \left(1-\alpha \right)\left(2.15-3.93\alpha +2.7\alpha^{2}\right)}{\left(1+2\alpha \right){\left(1-\alpha \right)}^{3/2}},\;\alpha =\frac{a_{c}}{h}$$

where $K_{IC}^{S}$ is the unstable fracture toughness (MPa·m^1/2^); $F_{max}$ represents the peak load (kN); and $a_{c}$ represents the critical effective crack length as expressed in Equation (5).

(5) $$a_{c}=\frac{2}{\pi }\left(h+h_{0}\right)\arctan\sqrt[{0}]{\frac{tEV_{c}}{32.6F_{max}}-0.1135}-h_{0}$$

where $h_{0}$ is the thickness of the clamped extensometer (0.001 m); t represents the specimen width (0.1 m); $V_{c}$ represents the critical CMOD (μm); and E represents the calculated elastic modulus as expressed in Equation (6).

(6) $$E=\frac{1}{tc_{i}}\left[3.7+32.6\tan^{2}\left(\frac{\pi }{2}\frac{a_{0}+h_{0}}{h+h_{0}}\right)\right]$$

where $c_{i}$ is the CMOD/load of linear range.

In this study, nonstandard specimens were used for the test, and the results of the fracture properties test need to be transformed in Equation (7).

(7) $$K_{IC}^{S}=\sqrt[{\alpha }]{\frac{V_{NS}}{V_{S}}}\sqrt{\frac{h_{S}}{h_{NS}}}K_{IC}^{NS}\left(h\leq 750\mathrm{mm}\right)$$

where subscripts S and NS represent the standard and nonstandard specimens, respectively; V is the specimen volumes (m^3^); h is the height of specimens (m); K is the fracture toughness (MPa·m^1/2^); and α represents the parameter of Weibull (α = 10).

According to the double-K fracture theory, the fracture state can be judged by determining the following relations: If $K_{I}$ < $K_{IC}^{Q}$, the cracks will not spread; If $K_{I}$ = $K_{IC}^{Q}$, the cracks begin to spread; If $K_{IC}^{Q}$ < $K_{I}$ < $K_{IC}^{S}$, the crack is in a stable expansion stage; If $K_{I}$ > $K_{IC}^{S}$, the cracks spread unsteadily.

Fracture energy $G_{F}$ represents the work down per unit area along the crack propagation direction per unit length. As a fracture parameter, which can represent the energy consumed by fracture propagation, it can be calculated by Equations (8) and (9) as follows:

(8) $$G_{F}=\frac{W_{0}}{A}+\frac{mg\delta_{0}}{A},\;m=m_{1}+m_{2}$$

(9) $$\;A=b\left(h-a_{0}\right)$$

where $W_{0}$ is the area under the load-mid-span deflection curve (N·m); A represents the area of the fracture part (m^2^); $m_{1}$ and $m_{2}$ are the mass of the specimen on the support span and the loading device, respectively (kg); and $\delta_{0}$ represents mid-span deflection of a specimen when it finally fractures (m).

### 2.5. Microscopic Test

In order to accurately observe the micromorphology of MFA-GPC and reveal the effect of MFA on the hydration products of MFA-GPC, the SEM produced by Zeiss Company in Germany was used to analyze the micromorphology. In order to accurately analyze the phase composition of MFA-GPC, a Smart Lab Intelligent XRD instrument produced by Neo-Science Electric Co., LTD.(Shimane, Japan) was used in this study. Samples were first crushed, ground and soaked in anhydrous ethanol, and then the powder sifted through a 45 μm sieve was selected as the test samples. The scanning angle range of the samples was 5°~80°, and the scanning time of each point was 0.2 s. FTIR analysis is a common technique to study the molecular and chemical composition of cement-based materials and geopolymer concrete. In this study, a Nicolet IS5 Fourier transform infrared spectroscopy instrument produced by Thermo Fisher Scientific Co., Ltd. (Waltham, MA, USA) was used to conduct infrared spectral analysis on the MFA-GPC samples.

### 2.6. Toxic Leaching Test

Referring to the Chinese Standard (HJ/T 299-2007) [60], the extractant was prepared firstly. In addition, the 100 g MFA sample was weighed and placed in a dry container with a constant weight, which was dried at 105 °C. Then, 200 g MFA with a dry basis weight was weighed and placed in a 2 L extraction bottle. According to the water content of MFA, the volume of the extractant was calculated according to the liquid–solid ratio of 10:1 (L/kg) firstly. Secondly, the extractant was added and the bottle was fixed on the turnover vibration device with the rotating speed set to 30 R/min and the amplitude to 40 mm. Then, the extraction bottle was removed after shaking at room temperature for 20 h, and the leaching solution was collected with a pressure filter. The leaching solution was digested by referring to the Chinese standard GB 5085.3-2007 [61]. Finally, the digested leaching solution was analyzed and determined by ICP-AES. The leaching content of MFA-GPC heavy metals was also determined according to Chinese Standards HJ/T 299-2007 [60] and GB 5085.3-2007 [61]. The MFA-GPC specimens were broken and internal slurry particles were grinded until the size of the particles was less than 9.5 mm. ICP-AES was used to analyze and determine the concentration of heavy metals in the leaching solution of MFA-GPC specimens.

## 3. Results and Discussion

### 3.1. Compressive Strength

**Figure 6:**
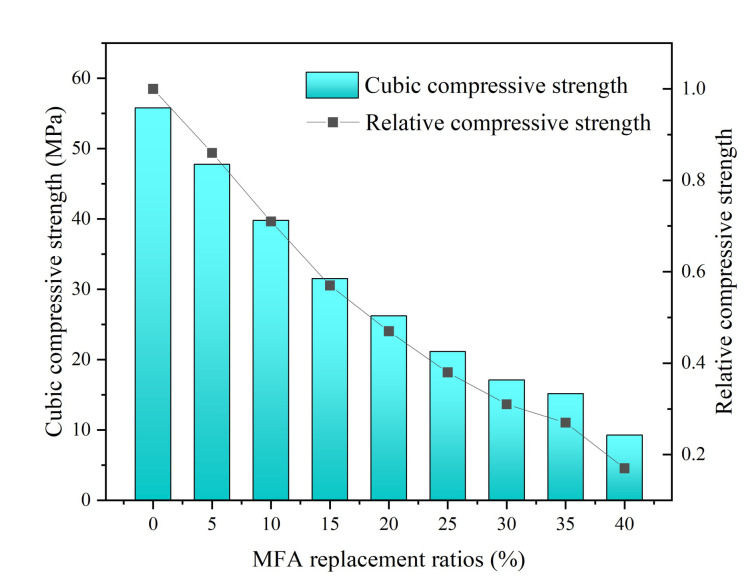
Cubic compressive strength versus MFA replacement ratios.

As shown in Figure 6, the compressive strength of MFA-GPC decreased gradually with the increase of the MFA replacement ratio. When the MFA replacement ratio was less than 15%, the compressive strength of MFA-GPC decreased approximately linearly with the increase of the MFA replacement ratio, and then decreased gradually with the increase of the MFA replacement ratio. It can be revealed that the compressive strength of MFA-GPC reached 26.24 MPa even when the replacement rate of MFA increased to 20%. When the MFA content was increased to 40% of the maximum, the compressive strength of MFA-GPC decreased sharply, to only 17% of that of the reference group.

The cubic compressive strength gradually decreasing with the increasing MFA replacement ratio was also reported in previous studies [62,63]. Yang et al. [63] proposed that the substitution rate of MFA for cement in cement-based materials should be controlled within 40% to ensure the basic strength. The reason may be that CaO is the main component with gelling activity in MFA, while less AlO_3_ and SiO_2_ result in weaker gelling activity. This is unlike fly ash and metakaolin, which are mainly composed of AlO_3_ and SiO_2_. Some research [47] reported that the content of Si and Al in the matrix decreased accordingly, and more C(-A) -S-H and N-A-S-H gels cannot be produced, which indicated that the compressive strength of MFA-based materials gradually decreased. In the study by Jiang et al. [64], adding calcium aluminate cement to the MFA-based geopolymer increased the aluminum content in the matrix. They found that the geopolymer with 30% calcium aluminate cement had a dense hydrogel structure and high compressive strength, which indicated that the content of silicon and aluminum in the matrix had a greater influence on the hydrogel products and strength of MFA-GPC.

**Figure 7:**
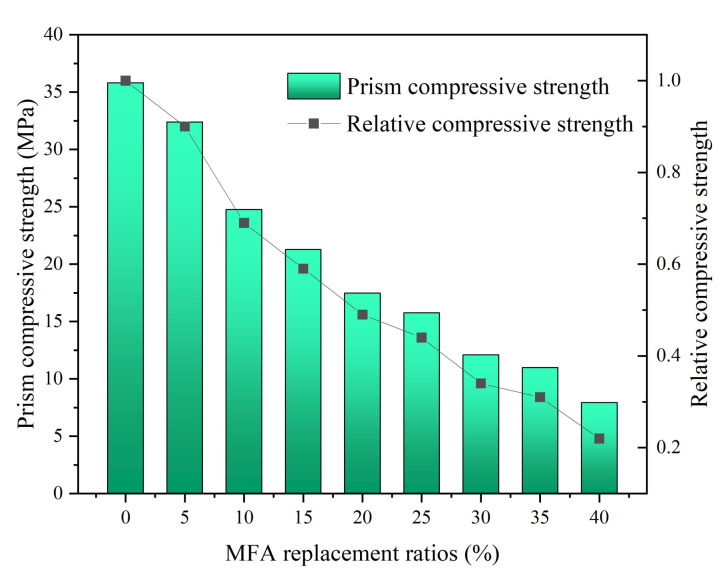
Prism compressive strength versus MFA replacement ratios.

As presented in Figure 7, the prism compressive strength of MFA-GPC decreased gradually with the increase of the MFA replacement ratio. When the MFA replacement ratio reached 10%, the prism compressive strength decreased by 31% compared with that of the reference group. As the MFA replacement ratio continued to increase to 40%, the prism compressive strength rapidly decreased to 7.94 MPa, which is 78% lower than that of the reference group, which is basically similar to the influence of the MFA replacement ratio on cubic compressive strength.

### 3.2. Splitting Tensile Strength

**Figure 8:**
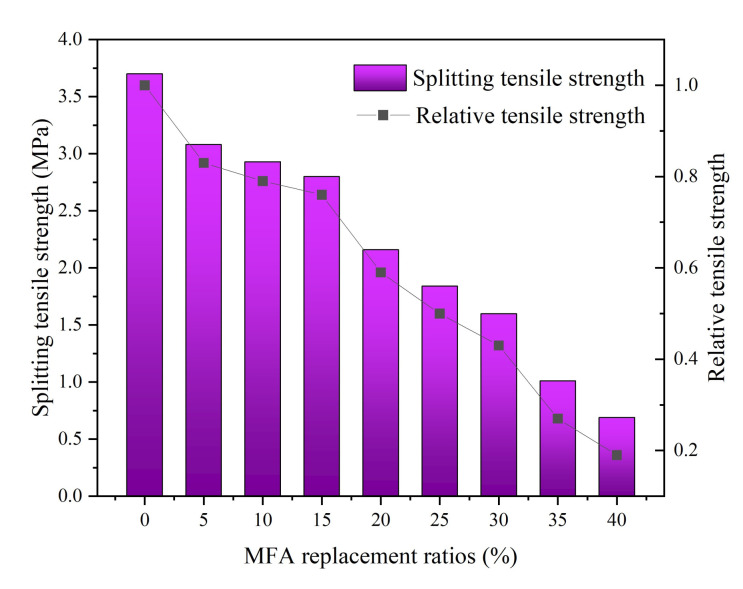
Splitting tensile strength versus MFA replacement ratios.

The tensile properties of MFA-GPC gradually decreased with the increase of MFA replacement ratio (Figure 8). When the MFA replacement rates were 5%, 10%, 15%, 20%, 25%, 30% and 35%, the splitting tensile strength of the specimens decreased by 16.76%, 20.81%, 24.32%, 41.62%, 50.27%, 56.76% and 72.70%, respectively, compared with the reference group. It can be seen that when the MFA replacement ratio was 5%, 20% and 35%, the splitting tensile strength decreased significantly, reaching 17%, 17% and 16%, respectively, compared with the previous group. As the MFA replacement ratio continued to increase to 40%, the splitting tensile strength also decreased to 0.69 MPa, which is 81% lower than the reference group.

In addition to the fact that a lack of silicon and aluminum in MFA decreases the strength of MFA-GPC, it may also be due to the high chloride contents of MFA. It has been pointed out that some chloride ions reduce the service life of reinforced concrete structures by interacting with hydration products to form Friedel salts (C_3_A·CaCl_2_·10H_2_O) [65]. A similar result was also reported by Singh et al. [66]: they found that the splitting tensile strength of mixed specimens gradually decreased with the increasing of MFA content. Increasing the replacement ratio of MFA will result in the addition of large amounts of chloride ions, which increases the curvature of the internal pores and the porosity of MFA-GPC, thus reducing the splitting tensile strength of MFA-GPC.

### 3.3. Elastic Modulus

**Figure 9:**
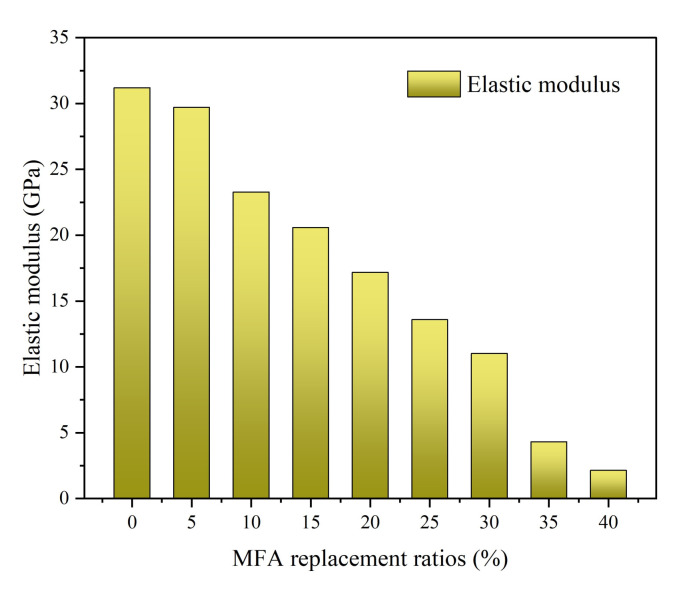
Elastic modulus versus MFA replacement ratios.

As shown in Figure 9, the elastic modulus of MFA-GPC decreased with the increase of the MFA replacement ratio, and the overall trend was consistent with the prism compressive strength. The elastic modulus of the specimens with a 5% MFA replacement rate did not change evidently compared with the reference group. When the MFA substitution rates were 10%, 15%, 20%, 25%, 30% and 35%, the elastic modulus of the specimens decreased by 25.38%, 34.01%, 44.94%, 56.44%, 64.65% and 86.15% compared with the baseline group, respectively. The elastic modulus of samples with an MFA replacement ratio of 10% and 35% decreased significantly. As the MFA replacement ratio continued to increase to 40%, the elastic modulus of the specimens decreased to 2.15 GPa, and decreased by 93% compared with the baseline group.

Guo et al. [67] studied the addition of tobermorite to MFA, and found the elastic modulus of tobermorite was reduced by blocking the synthesis of silicon tetrahedral chains. When the replacement ratio of MFA is low, the overall structure and hydration products of MFA-GPC are not significantly changed compared with GPC, and still have a relatively dense three-dimensional network gel structure [62], so it has a good structural stability. However, the internal structure will become unconsolidated and the overall structural stability will be reduced with the increasing replacement ratios of MFA, which will lead to the decrease of the deformation resistance of MFA-GPC. Therefore, when the MFA replacement ratio was 35% and 40%, the elastic modulus of MFA-GPC decreased greatly.

### 3.4. Fracture Properties

**Figure 10:**
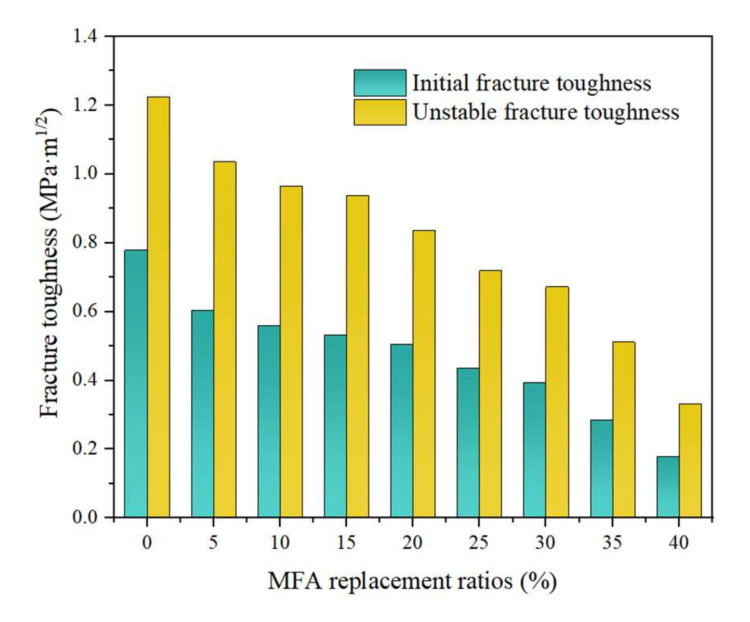
Initial and unstable fracture toughness with different MFA replacement ratios.

The effect of the MFA replacement rate on the fracture toughness of MFA-GPC is shown in Figure 10. It can be seen that the initiation fracture toughness and unstable fracture toughness of the reference group were 0.801 MPa·m^1/2^ and 1.260 MPa·m^1/2^, respectively. The fracture toughness of MFA-GPC decreased gradually with the increase of the MFA substitution rate. When the MFA replacement rates were 5%, 10%, 15%, 20%, 25%, 30% and 35%, the initiation fracture toughness of the specimens decreased by 22.35%, 28.09%, 29.96%, 43.95%, 49.44% and 63.30%, respectively, compared with the reference group. The unstable fracture toughness decreased by 15.48%, 23.49%, 28.97%, 31.67%, 41.27%, 45.08% and 58.25%, respectively. When the MFA replacement rate was 40%, the fracture toughness of the specimens appeared to be the minimum, and the initiation fracture toughness and unstable fracture toughness reduced by 77.03% and 72.86%, respectively, compared with the reference group.

Due to the addition of MFA, the internal structure of GPC becomes loose and the original defects of MFA-GPC become more and more. This means that the size and number of original cracks in MFA-GPC increase, and the bearing capacity of MFA-GPC decreases, which is not conducive to preventing the generation and propagation of cracks. Therefore, the fracture toughness of MFA-GPC decreases with the increase of the MFA replacement ratio.

**Figure 11:**
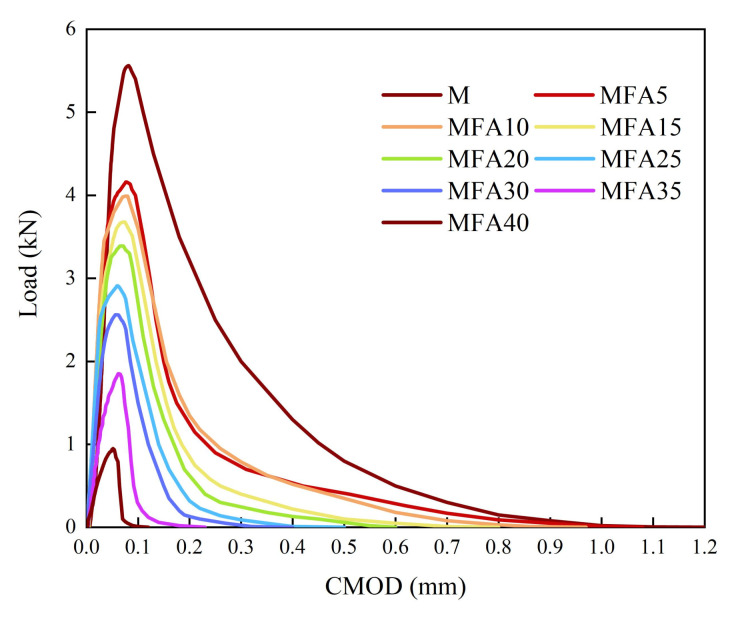
Load—CMOD curves.

Figure 11 shows the P-CMOD curves of MFA-GPC at different MFA replacement rates. It can be revealed from Figure 11 that MFA has a significant effect on the P-CMOD curve of specimens. As the replacement ratio of MFA increases, the peak load of the specimen decreases gradually as a whole, and the CMOD corresponding to the peak load decreases gradually. The plumpness of the P-CMOD curve decreases with the increase of the MFA replacement rate. As the substitution rate increases, the descent section of the curve becomes steeper and the tail of the curve becomes shorter. With the increase of the MFA replacement rate, the brittleness of MFA-GPC increases, which results in the development of cracks that cannot be effectively prevented.

**Figure 12:**
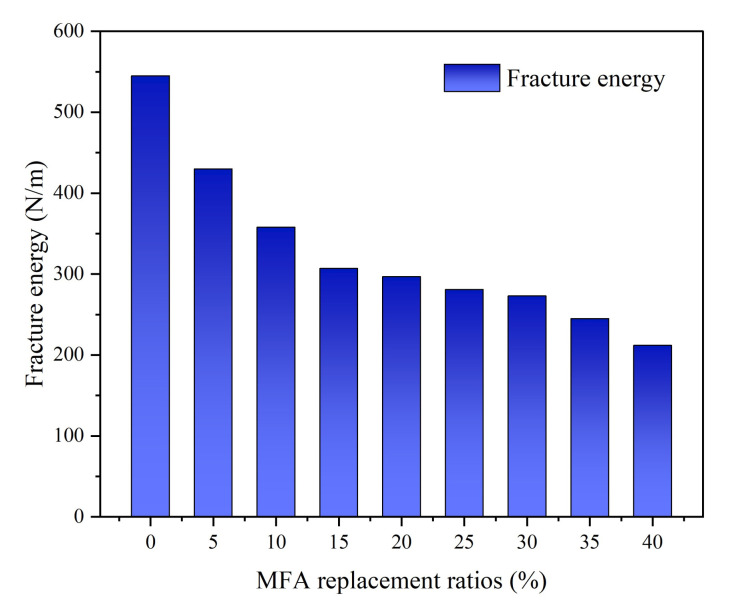
Fracture energy versus MFA replacement ratios.

The effect of the MFA replacement rate on the fracture energy of MFA-GPC is shown in Figure 12. As shown, the fracture energy of MFA-GPC decreased with the replacement rate of MFA, and the overall change trend was similar to that of fracture toughness. It is obvious that only a 5% MFA substitution still had a significant effect on the fracture energy. The fracture energy of the reference group was 545 N/m. When 5% MFA was added, the fracture energy of the specimen decreased to 430 N/m, which is 21.09%. The fracture energy of the specimens with 10% and 15% MFA substitution also decreased significantly by 34.20% and 43.71%, respectively. As the replacement rate of MFA continued to increase, the decrease in fracture energy of specimens eased down gradually. When the MFA replacement rate was 40%, the fracture energy of the specimens reached the minimum value, which was 61.09% lower than that of the reference group.

The addition of MFA reduces the compactness and strength of MFA-GPC, thus reducing the resistance of MFA-GPC to the development and propagation of micro-cracks. The cementing strength of coarse aggregates and geopolymer pastes in MFA-GPC decreases further with the increase of the MFA replacement rate. Cracks develop steadily in a less energy-consuming manner and do not penetrate the coarse aggregates of the entire fracture surface, but rather more through the relatively weak bonds between the geopolymer pastes and the coarse aggregates.

In addition, heavy metal ions in MFA are added to the matrix. Heavy metal ions form hydroxyl complexes with hydroxide ions in the reaction system of in situ polymers, resulting in a lower alkalinity and higher viscosity of in situ polymers, which hinder the dissolution of silica-aluminum oxide. At the same time, heavy metal hydroxy complex ions will interfere with the condensation reaction of the silicon/aluminum oxide tetrahedron, further hindering the growth of the silica-aluminum network framework and reducing the gel structure strength of the system, which will lead to the decrease of MFA-GPC strength [68]. When the substitution rate of MFA is more than 10%, a large amount of CaO in MFA will form calcium aluminate hydrate (C-A-H) by alkali excitation and produce certain strength alunite (Aft) by reacting with SO^4+^, which can compensate for the strength loss caused by the reduction of calcium silicate aluminate (C-A-S-H) gel to a certain extent [69]. Therefore, when the replacement rate of MFA is more than 10%, the decrease of MFA-GPC fracture energy tends to be gentle gradually.

### 3.5. Microanalysis of MFA-GPC

#### 3.5.1. SEM Analysis

**Figure 13:**
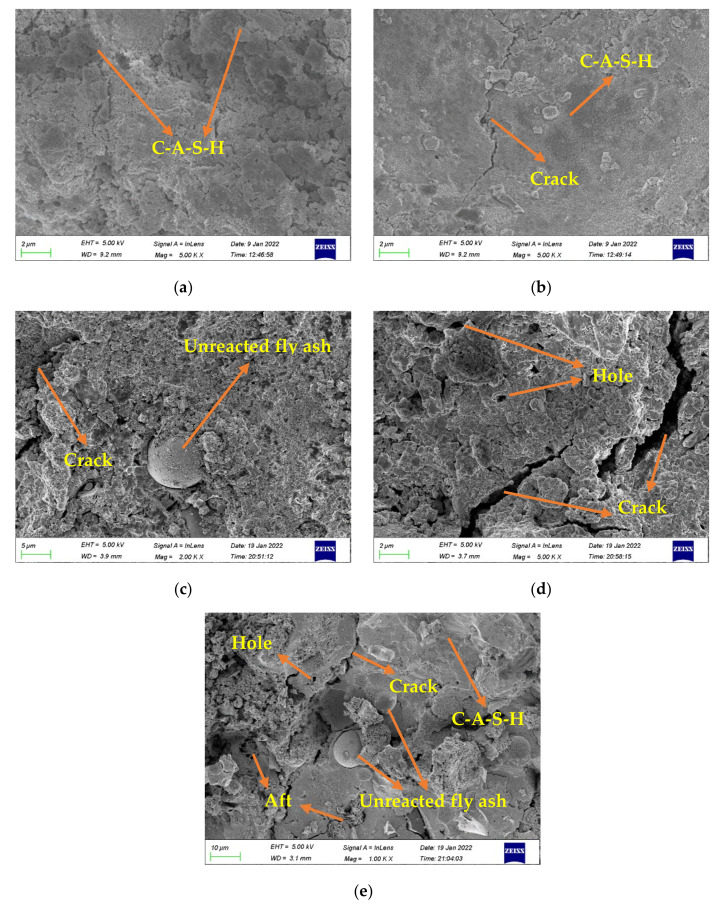
SEM images of fractured surface for specimens: (**a**) M, (**b**) MFA10, (**c**) MFA20, (**d**) MFA30 and (**e**) MFA40.

Figure 13 presents the microstructure images of MFA-GPC with different MFA replacement rates. As can be seen from Figure 13a, unreacted fly ash particles, metakaolin, obvious cracks and holes are not found. The GPC without MFA has a continuous and dense silicate-aluminate gel structure. Figure 13b shows the microstructure of a specimen at an MFA substitution rate of 10%. It can be seen that the specimens are still mainly composed of a silicate-aluminate gel structure. The difference is that there are small cracks in the specimens with a 10% MFA substitution rate, but the macro-mechanical properties of the specimens are not greatly affected. This also explains to some extent why MFA-GPC specimens with low MFA replacement ratio (5~20%) still have a high strength. However, with the increase of the MFA substitution rate, it is obvious that cracks of different thicknesses begin to appear inside the specimens, accompanied by small holes, which will undoubtedly weaken the overall structural strength of the specimens. With the further increase of the MFA substitution rate, unreacted fly ash particles gradually increase; the internal structure of GPC becomes more porous; the crack width increases significantly; and even the continuous silica-aluminate gel cannot be observed in Figure 13e. This also means that there are more internal weaknesses, which further leads to the gradual decrease of MFA-GPC strength.

FA and MK in the reference group specimens have fully reacted and generated silica-aluminate gels such as C-A-S-H and N-A-S-H, depending on the degree of hydration reaction. Figure 13c shows that unreacted fly ash particles are encapsulated in silica-aluminate gels and a large amount of needle-rod crystals appears in the interior of the specimen. In combination with previous studies, it can be judged to be Aft [46,70]. Some scholars have indicated that Alt is produced by the reaction of sulfate ions carried by MFA with calcium aluminate hydrate [71]. The generated alunite fills the voids and cracks in the matrix and partially compensates for the strength loss caused by less silica-aluminate gels and the looseness of the matrix structure, which makes the MFA-GPC with a high MFA replacement rate (40%) still have a certain strength.

#### 3.5.2. XRD Analysis

**Figure 14:**
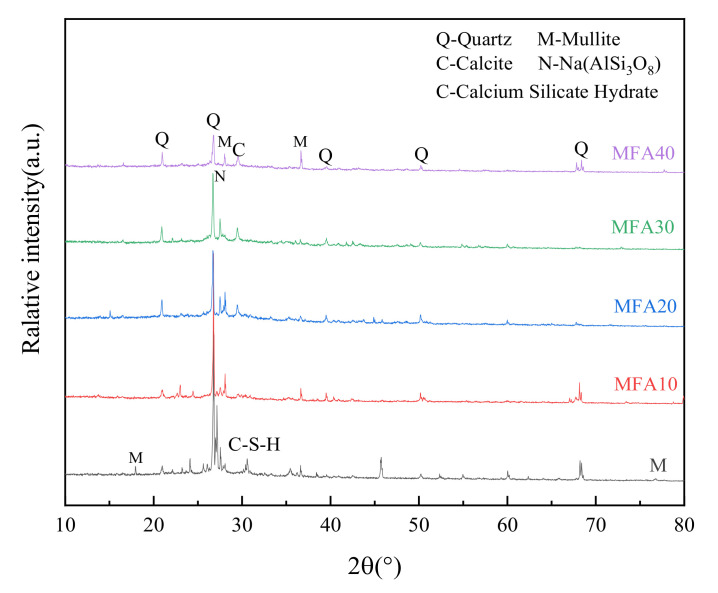
XRD patterns of MFA-GPC specimens.

XRD analysis results are shown in Figure 14 to accurately analyze the effect of MFA replacement rates on the phase of GPC. As shown in Figure 14, no apparent hump can be observed due to the high diffraction peak at 26.7 degrees. However, small diffraction peak packages do occur in the range of 18 to 36 degrees, which indicates that the main hydration products of MFA-GPC are still amorphous aluminosilicate minerals gels. It can be observed that with the increase of the MFA substitution rate, the diffraction peak packages in the range of 18 degrees to 36 degrees gradually decrease. Because the aluminum–silicon content of MFA is lower than the metakaolin content and because of the insufficient aluminum–silicon contents, the amorphous aluminum silicate and other hydration products in MFA-GPC are reduced correspondingly.

The samples tested by XRD were obtained from the crushed MFA-GPC, which contained river sand and other substances. Therefore, sharp diffraction peaks of quartz and mullite appear in MFA-GPC samples with different MFA substitution rates [72]. There is no obvious calcite diffraction peak in the XRD spectrum of the sample when the MFA substitution rate is low (10%). However, the calcite diffraction peak appears at the angle of 29° when the MFA substitution rate exceeds 20%. This shows that calcite is a new mineral with the increase of the MFA substitution rate, which means that the increase of the MFA substitution rate will aggravate the carbonation degree of the geopolymer concrete, and this can be verified through FTIR analysis.

Figure 14 shows that the hydration products of MFA-GPC samples are mainly amorphous aluminum silicate mineral phases and crystalline phases such as quartz and mullite, which have no obvious difference compared with the reference group samples. This is similar to Huang’s research results [53]. As the main support on the strength of geopolymer cementitious material, C-A-S-H gel gradually decreases as the MFA substitution rate increases, which explains that the mechanical strength of MFA-GPC decreases with the increase of the MFA replacement ratio from the perspective of microstructure. It is also important to note that, except for calcite, the hydration products of the MFA-GPC samples have no new mineral phases compared with the reference group samples, which indicates that heavy metals in the MFA may have been bonded to the geopolymer framework [73].

#### 3.5.3. FTIR Analysis

**Figure 15:**
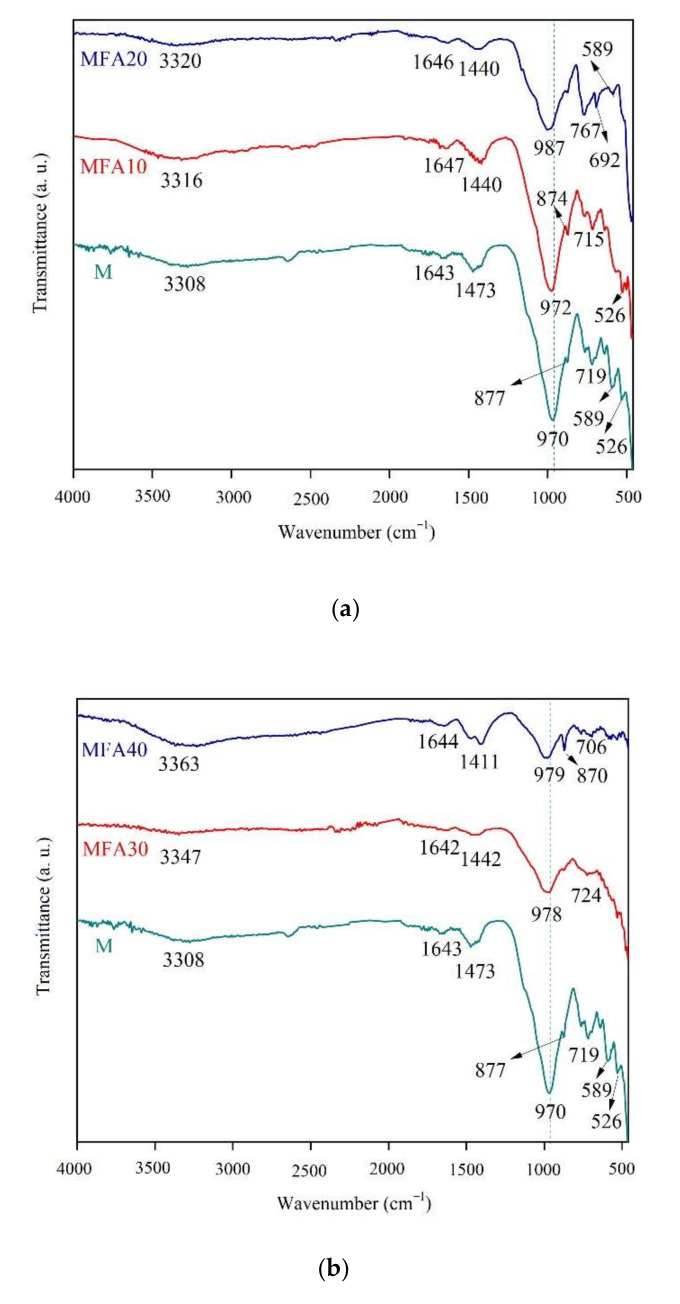
FTIR spectra of the MFA-GPC specimens: (**a**) MFA10 and MFA20 and (**b**) MFA30 and MFA40.

The FTIR test results of the MFA-GPC are shown in Figure 15. It can be seen that absorption peaks with different peak heights appear in each group of samples near 970 cm^−1^. This peak is generated by the symmetrical stretching vibration of Si-O in the Q_2_ tetrahedron, and it is also the main infrared absorption peak of C-A-S-H, which is the major cementitious component of geopolymer [69]. It can be observed that the infrared absorption peak height of the C-A-S-H gel of the sample doped with MFA decreases significantly. When the MFA substitution rate is 40%, the peak height decreases to the lowest, which indicates that the amount of gels decreases with the increase of the MFA substitution rate, and the decrease of C-A-S-H gel will further affect the mechanical properties of MFA-GPC.

Absorption peaks formed by the out-of-plane bending vibration of Si-O and deformation vibration of Si-O-Al are 526 cm^−1^ and 589 cm^−1^, respectively. The content of Si and Al in the specimens is insufficient with the increase of the MFA substitution rate, which leads to the disappearance of these two absorption peaks. The absorption peak formed by C-O symmetrical telescopic vibration in CaCO_3_ is located in the wavenumber of 692~719 cm^−1^ and 1411~1473 cm^−1^, which indicates that the specimen may be carbonized to produce CaCO_3_ [74]. It can be seen that the absorption peak height formed by the C-O symmetrical telescopic vibration increases significantly when the MFA substitution rate is 40%. The peak height and area of the corresponding absorption peak can be used as a basis for the degree of carbonization of samples, which means that the degree of carbonization of samples with 40% MFA substitution increases significantly. The increase of the MFA substitution rate increases the calcium contents in the matrix and leads to the easy carbonization of CO_2_ in the air with the specimen [75]. The absorption peaks of 1646~1653 cm^−1^ and 3356~3398 cm^−1^ are formed by the flexural and extensional vibrations of the H-O-H group in the H_2_O molecule, respectively. It can be seen that when the MFA substitution rate is 10%, 20% and 30%, the peak height of absorption caused by the H_2_O molecule is close to that of the reference group. When the substitution rate is 40%, the peak height is slightly higher than that of the reference group. This indicates that the free water content in the sample does not change significantly compared with the reference group when the MFA replacement rate is less than 40%. When the MFA substitution rate reaches 40%, similar to the previous study, the free water content in the specimen gradually exceeds the reference group, but the overall change trend is not significant [53].

#### 3.5.4. Toxic Leaching Analysis

An environmental risk assessment of MFA-GPC must be carried out to meet the requirements of large-scale engineering applications. It should be ensured that it does not pollute the environment due to the high leaching content of heavy metals. The results of the MFA and MFA-GPC toxic leaching analysis are shown in Table 4. Similar to the previous research results [49,50], it is obvious that the leaching concentration of heavy metals in MFA-GPC is much lower than that of MFA and is below the standard limits.

## 4. Conclusions

In this study, the effect of MFA replacement ratios on the basic mechanical properties and fracture performance of MFA-GPC was investigated. At the same time, the influence of MFA on the microstructure of MFA-GPC was analyzed by combining the SEM test, XRD analysis and FTIR analysis. The ICP-AES test was employed to determine the leaching concentration of heavy metals in MFA-GPC. The following conclusions can be drawn:

(1) The basic mechanical properties of MFA-GPC show a linear downward trend with the continuous increase of MFA replacement ratios. When the MFA replacement rate reaches 40%, the cubic compressive strength, splitting tensile strength, axial compressive strength and elastic modulus decrease to 83%, 81%, 78%, and 93%, respectively compared with the reference group.
(2) The fracture properties of MFA-GPC gradually decrease with the increase of MFA replacement ratios. When the MFA replacement rate reaches 40%, the peak load, initiation fracture toughness, unstable fracture toughness and fracture energy are reduced by 83%, 77%, 73% and 61%, respectively, compared with the reference group.
(3) The microstructure of MFA-GPC becomes porous and loose with the addition of MFA. With the increase of MFA substitution ratios, voids and cracks in the matrix structure further increase, which explains the difference of mechanical properties between MFA-GPC and GPC. XRD and FTIR results indicate that the main hydration products of MFA-GPC are amorphous aluminosilicate minerals gels. However, the aluminum silicate gel products in MFA-GPC gradually decrease, and the degree of carbonization gradually increases.
(4) The leaching results of heavy metals from MFA-GPC show that the leaching content of each heavy metal in MFA-GPC is low compared with the standard limit, which indicates that MFA-GPC has an excellent fixation effect on heavy metals in MFA, and heavy metals are immobilized through physical immobilization and chemical bonding.
(5) In this study, the internal composition of GPC was assumed to be the same as that of OPC concrete, only one type of MFA was utilized and only Chinese specifications were referenced for the toxic leaching test, which brought certain limitations. In the future, the influence of diverse MFA can be studied according to the different internal composition in GPC and OPCC, and the international standards can be further referenced.

## Figures and Tables

**Table 1:** Chemical compositions of MFA.

Chemical Compositions (wt.%)	CaO	Cl	Na_2_O	K_2_O	SO_3_	SiO_2_	MgO	Al_2_O_3_	P_2_O_5_	Fe_2_O_3_
MK	35.87	24.50	15.37	9.42	8.19	3.19	1.71	0.72	0.52	0.34

**Table 2:** Chemical compositions of FA and MK.

Chemical Compositions (wt.%)	SiO_2_	Al_2_O_3_	Fe_2_O_3_	CaO + MgO	K_2_O + Na_2_O	SO_3_
FA	60.98	24.47	6.70	5.58	–	0.27
MK	54	43	≤1.3	≤0.8	≤0.7	–

**Table 3:** Mix proportions of MFA-GPC.

Mix ID	FA	MK	MFA	Alkali Activator	River Sand	Coarse Aggregate	Water-Reducing Agent
	kg/m^3^	kg/m^3^	%	kg/m^3^	kg/m^3^	kg/m^3^	%
M	234.9	234.9	0	371.7	577	1072	3.0
MFA5	234.9	211.4	5	371.7	577	1072	2.5
MFA10	234.9	187.9	10	371.7	577	1072	2.0
MFA15	234.9	164.4	15	371.7	577	1072	1.5
MFA20	234.9	141.2	20	371.7	577	1072	1.0
MFA25	234.9	117.5	25	371.7	577	1072	0.5
MFA30	234.9	93.7	30	371.7	577	1072	0
MFA35	234.9	70.5	35	371.7	577	1072	0
MFA40	234.9	47.0	40	371.7	577	1072	0

**Table 4:** Results of heavy metal leaching of MFA and MFA-GPC.

Heavy Metals	Standard A (mg/L)	Standard B (mg/L)	Standard C (mg/L)	MFA (mg/L)	MFA-GPC (mg/L)
Zn	120	100	100	4.71	0.70
Cu	120	100	100	0.67	0.07
Cr	15	15	4.5	0.06	0.03
Pb	1.2	5	0.25	1.48	0.11
Cd	0.6	1	0.15	0.19	-

## References

[1] Xie J.H., Chen W., Wang J.J., Fang C., Zhang B.X., Liu F. (2019). Coupling effects of recycled aggregate and GGBS/metakaolin on physicochemical properties of geopolymer concrete. Constr. Build. Mater..

[2] Wang L., Guo F.X., Yang H.M., Wang Y. (2021). Comparison of fly ash, PVA fiber, MgO and shrinkage-reducing admixture on the frost resistance of face slab concrete via pore structural and fractal analysis. Fractals.

[3] Kan L.L., Zhang L., Zhao Y.J., Wu M. (2020). Properties of polyvinyl alcohol fiber reinforced fly ash based Engineered Geopolymer Composites with zeolite replacement. Constr. Build. Mater..

[4] Wang L., Lu X., Liu L., Xiao J., Zhang G., Guo F., Li L. (2022). Influence of MgO on the hydration and shrinkage behavior of low heat Portland cement-based materials via pore structural and fractal analysis. Fractal Fract..

[5] Zhang P., Zhang H., Cui G., Yue X., Guo J., Hui D. (2021). Effect of steel fiber on impact resistance and durability of concrete containing nano-SiO_2_. Nanotechnol. Rev..

[6] Bouaissi A., Li L.Y., Abdullah M.M.A.B., Bui Q.B. (2019). Mechanical properties and microstructure analysis of FA-GGBS-HMNS based geopolymer concrete. Constr. Build. Mater..

[7] Wang L., Luo R., Zhang W., Jin M., Tang S. (2021). Effects of fineness and content of phosphorus slag on cement hydration, permeability, pore structure and fractal dimension of concrete. Fractals.

[8] Zhang X.M., Zhang P., Wang T.Y., Zheng Y., Qiu L.H., Sun S.W. (2022). Compressive strength and anti-chloride ion penetration assessment of geopolymer mortar merging PVA fiber and nano-SiO_2_ using RBF–BP composite neural network. Nanotechnol. Rev..

[9] Golewski G.L. (2018). Green concrete composite incorporating fly ash with high strength and fracture toughness. J. Clean. Prod..

[10] National Bureau of Statistics of China (2022). Operation of Building Materials Industry in 2021.

[11] Luhar S., Chaudhary S., Luhar I. (2019). Development of rubberized geopolymer concrete: Strength and durability studies. Constr. Build. Mater..

[12] Assi L.N., Deaver E.E., Ziehl P. (2018). Effect of source and particle size distribution on the mechanical and microstructural properties of fly Ash-Based geopolymer concrete. Constr. Build. Mater..

[13] Zhang P., Kang L., Zheng Y., Zhang T., Zhang B. (2022). Influence of SiO_2_/Na_2_O molar ratio on mechanical properties and durability of metakaolin-fly ash blend alkali-activated sustainable mortar incorporating manufactured sand. J. Mater. Res. Technol..

[14] Zhang N., Yan C.Y., Li L., Khan M. (2022). Assessment of fiber factor for the fracture toughness of polyethylene fiber reinforced geopolymer. Constr. Build. Mater..

[15] Liew Y.M., Heah C.Y., Mohd M.A.B., Kamarudin H. (2016). Structure and properties of clay-based geopolymer cements: A review. Prog. Mater. Sci..

[16] Abadel A.A., Albidah A.S., Altheeb A.H., Alrshoudi F.A., Abbas H., Salloum Y.A.A. (2021). Effect of molar ratios on strength, microstructure & embodied energy of metakaolin geopolymer. Adv. Concr. Constr..

[17] Gao Z., Zhang P., Wang J., Wang K., Zhang T. (2022). Interfacial properties of geopolymer mortar and concrete substrate: Effect of polyvinyl alcohol fiber and nano-SiO_2_ contents. Constr. Build. Mater..

[18] Jing Y., Han Q., Zhang P., Wu J., Zhang D., Zhang T. (2022). Comprehensive review of the properties of fly ash-based geopolymer with additive of nano-SiO_2_. Nanotechnol. Rev..

[19] Zhang P., Wang K., Wang J., Guo J., Ling Y. (2021). Macroscopic and microscopic analyses on mechanical performance of metakaolin/fly ash based geopolymer mortar. J. Clean. Prod..

[20] Zhang P., Wang K.X., Wang J., Guo J.J., Hu S.W., Ling Y.F. (2020). Mechanical properties and prediction of fracture parameters of geopolymer/alkali-activated mortar modified with PVA fiber and nano-SiO_2_. Ceram. Int..

[21] Gao Z., Zhang P., Guo J.J., Wang K.X. (2021). Bonding behavior of concrete matrix and alkali-activated mortar incorporating nano-SiO_2_ and polyvinyl alcohol fiber: Theoretical analysis and prediction model. Ceram. Int..

[22] Zhang P., Han X., Zheng Y.X., Wan J.Y., Hui D. (2021). Effect of PVA fiber on mechanical properties of fly ash-based geopolymer concrete. Rev. Adv. Mater. Sci..

[23] Wang K.X., Zhang P., Guo J.J., Gao Z. (2021). Single and synergistic enhancement on durability of geopolymer mortar by polyvinyl alcohol fiber and nano-SiO_2_. J. Mater. Res. Technol..

[24] Huang X., Huang T., Li S., Muhammad F., Xu G., Zhao Z., Yu L., Yan Y., Li D., Jiao B. (2016). Immobilization of chromite ore processing residue with alkali-activated blast furnace slag-based geopolymer. Ceram. Int..

[25] Hassan A., Arif M., Shariq M. (2019). Use of geopolymer concrete for a cleaner and sustainable environment—A review of mechanical properties and microstructure. J. Clean. Prod..

[26] Noushini A., Aslani F., Castel A., Gilbert R.I., Uy B., Foster S. (2016). Compressive stress-strain model for low-calcium fly ash-based geopolymer and heat-cured Portland cement concrete. Cem. Concr. Comp..

[27] Yan J.H., Chen T., Li X.D., Zhang J., Lu S.Y., Ni M.J., Cen K.F. (2006). Evaluation of PCDD/Fs emission from fluidized bed incinerators co-firing MSW with coal in China. J. Hazard. Mater..

[28] Ye N., Chen Y., Yang J., Liang S., Hu Y., Xiao B., Huang Q., Shi Y., Hu J., Wu X. (2016). Co-disposal of MSWI fly ash and Bayer red mud using an one-part geopolymeric system. J. Hazard. Mater..

[29] Girskas G., Kizinievič O., Kizinievič V. (2021). Analysis of durability (frost resistance) of MSWI fly ash modified cement composites. Arch. Civ. Mech. Eng..

[30] Hjelmar O. (1996). Disposal strategies for municipal solid waste incineration residues. J. Hazard. Mater..

[31] Shi H., Kan L. (2009). Leaching behavior of heavy metals from municipal solid wastes incineration (MSWI) fly ash used in concrete. J. Hazard. Mater..

[32] Tan W.F., Lv J.W., Deng Q.W., Zhang X.W. (2015). Application of a combination of municipal solid waste incineration fly ash and lightweight aggregate in concrete. J. Adhes. Sci. Technol..

[33] Wang L., Zhang Y., Chen L., Guo B., Tan Y., Sasaki K., Tsang D.C.W. (2022). Designing novel magnesium oxysulfate cement for stabilization/solidification of municipal solid waste incineration fly ash. J. Hazard. Mater..

[34] Li Z.G., Kondoa R., Ko I. (2021). Development of foamed geopolymer with addition of municipal solid waste incineration fly ash. J. Adv. Concr. Technol..

[35] Charles H.K., Alvin W.M., Barford J.P., McKay G. (2010). Use of Incineration MSW Ash: A Review. Sustainability.

[36] Quina M.J., Bordado J.C., Quinta F.R.M. (2008). Treatment and use of air pollution control residues from MSW incineration: An overview. Waste Manag..

[37] Lee W.K., Park E.Z., Kim Y.D., Son S.G., Lee J.H. (2008). Development of Inorganic Binder with MSWI Ash. Mater. Sci. Forum..

[38] Guo X., Shi H., Wu K., Ju Z., Dick W.A. (2015). Performance and risk assessment of alinite cement-based materials from municipal solid waste incineration fly ash (MSWIFA). Mater. Struct..

[39] Nishida K., Nagayoshi Y., Ota H., Nagasawa H. (2001). Melting and stone production using MSW incinerated ash. Waste Manag..

[40] Mangialardi T. (2001). Sintering of MSW fly ash for reuse as a concrete aggregate. J. Hazard. Mater..

[41] Sakai S., Hiraoka M. (2000). Municipal solid waste incinerator residue recycling by thermal processes. Waste Manag..

[42] Polettini A., Pomi R., Trinci L., Muntoni A., Lo M.S. (2004). Engineering and environmental properties of thermally treated mixtures containing MSWI fly ash and low-cost additives. Chemosphere.

[43] Hong K.J., Tokunaga S., Ishigami Y., Kajiuchi T. (2000). Extraction of heavy metals from MSW incinerator fly ash using saponins. Chemosphere.

[44] Saikia N., Kato S., Kojima T. (2007). Production of cement clinkers from municipal solid waste incineration (MSWI) fly ash. Waste Manag..

[45] Zheng L., Wang W., Gao X. (2016). Solidification and immobilization of MSWI fly ash through aluminate geopolymerization: Based on partial charge model analysis. Waste Manag..

[46] Liu J., Hu L., Tang L., Ren J. (2021). Utilisation of municipal solid waste incinerator (MSWI) fly ash with metakaolin for preparation of alkali-activated cementitious material. J. Hazard. Mater..

[47] Ren J., Hu L., Dong Z., Tang L., Xing F., Liu J. (2021). Effect of silica fume on the mechanical property and hydration characteristic of alkali-activated municipal solid waste incinerator (MSWI) fly ash. J. Clean. Prod..

[48] Tian X., Rao F., Leon P.C.A., Song S. (2020). Effects of aluminum on the expansion and microstructure of alkali-activated MSWI fly ash-based pastes. Chemosphere.

[49] Li Y., Min X., Ke Y., Liu D., Tang C. (2019). Preparation of red mud-based geopolymer materials from MSWI fly ash and red mud by mechanical activation. Waste Manag..

[50] Jin M., Zheng Z., Sun Y., Chen L., Jin Z. (2016). Resistance of metakaolin-MSWI fly ash based geopolymer to acid and alkaline environments. J. Non-Cryst. Solids..

[51] (2008). Concrete Admixtures.

[52] (2011). Specification for Mix Proportion Design of Ordinary Concrete.

[53] Jin M. (2011). Immobilization of Heavy Metals in Municipal Solid Waste Incineration (MSWI) Fly Ash with Geopolymer. Ph.D. Thesis.

[54] Zhang Y., Sun W., Sha J., Lin W., Zheng K., Liu S. (2003). Preparation, properties and mechanism of fly ash based geopolymer concrete. J. Build..

[55] (2019). Standard for Test Methods of Concrete Physical and Mechanical Properties.

[56] Zhang P., Wang J., Li Q., Wan J., Ling Y. (2021). Mechanical and fracture properties of steel fiber-reinforced geopolymer concrete. Sci. Eng. Compos. Mater..

[57] Chen C., Chen X.D., Guo S.S. (2019). Experimental study on acoustic emission characteristic of fatigue crack growth of self-compacting concrete. Struct. Control Health.

[58] Fornusek J., Tvarog M. (2014). Influence of Casting Direction on Fracture Energy of Fiber-Reinforced Cement Composites. Key Eng. Mater..

[59] (2005). Norm for Fracture Test of Hydraulic Concrete.

[60] (2007). Solid Waste-Extraction Procedure for Leaching Toxicity-Sulphuric Acid & Nitric Acid Method.

[61] (2007). Identification Standards for Hazardous Wastes—Identification for Extraction Toxicity.

[62] Lenormand T., Rozière E., Loukili A., Staquet S. (2015). Incorporation of treated municipal solid waste incineration electrostatic precipitator fly ash as partial replacement of Portland cement: Effect on early age behaviour and mechanical properties. Constr. Build. Mater..

[63] Yang Z., Ji R., Liu L., Wang X., Zhang Z. (2018). Recycling of municipal solid waste incineration by-product for cement composites preparation. Constr. Build. Mater..

[64] Jiang G., Min X., Ke Y., Liang Y., Yan X., Xu W., Lin Z. (2022). Solidification/stabilization of highly toxic arsenic-alkali residue by MSWI fly ash-based cementitious material containing Friedel’s salt: Efficiency and mechanism. J. Hazard. Mater..

[65] Rémond S., Pimienta P., Bentz D.P. (2002). Effects of the incorporation of Municipal Solid Waste Incineration fly ash in cement pastes and mortars. Cem. Concr. Res..

[66] Singh D., Kumar A. (2017). Geo-environmental application of municipal solid waste incinerator ash stabilized with cement. J. Rock. Mech. Geotech..

[67] Guo X., Song M. (2018). Micro-nanostructures of tobermorite hydrothermal-synthesized from fly ash and municipal solid waste incineration fly ash. Constr. Build. Mater..

[68] Jaarsveld J.G.S., Deventer J.S.J. (1999). The effect of metal contaminants on the formation and properties of waste-based. Cem. Concr. Res..

[69] Wang Q., Kang S., Wu L., Tang N., Zhang Q. (2020). Molecular simulation of N-A-S-H and C-A-S-H in geopolymer cementitious system. J. Build. Mater..

[70] Yu H.T., Liu W.S., Beadham I., Deng Y., Hou H.B. (2013). A study into solidification of municipal solid waste incinerator fly ash in low-clinker slag cementitious materials. Fresen. Environ. Bull..

[71] Zhang W., Zhang J., Ye J., Wang H., Zhang J., Liu J. (2017). Influence of synthesis conditions on morphology of ettringite. J. Chin. Ceram. Soc..

[72] Pasupathy K., Berndt M., Sanjayan J., Rajeev P., Cheema D.S. (2017). Durability of low-calcium fly ash based geopolymer concrete culvert in a saline environment. Cem. Concr. Res..

[73] Xu J.Z., Zhou Y.L., Tang R.X. (2006). Study on the solidification of heavy metals by fly ash based geopolymers. J. Build. Mater..

[74] Smith B.C. (2018). Infrared Spectral Interpretation: A Systematic Approach.

[75] Chindaprasirt P., Jaturapitakkul C., Chalee W., Rattanasak U. (2009). Comparative study on the characteristics of fly ash and bottom ash geopolymers. Waste Manag..

[76] (2019). Standard for Pollution Control on the Hazardous Waste Landfill.

[77] (2008). Standard for Pollution Control on the Landfill Site of Municipal Solid Waste.

